# Determination of ^238^Pu, ^239+240^Pu and ^241^Am in air filters

**DOI:** 10.1007/s10967-016-5015-y

**Published:** 2016-09-03

**Authors:** Katarzyna Rzemek, Andrzej Czerwiński, Jakub Ośko, Katarzyna Tymińska, Małgorzata Dymecka, Tomasz Pliszczyński

**Affiliations:** 1 National Centre for Nuclear Research, Sołtana 7, 05-400 Otwock, Poland; 2 Faculty of Chemistry, University of Warsaw, Pasteura 1, 02-093 Warsaw, Poland

**Keywords:** Americium, Plutonium, Workplace monitoring, Air filters

## Abstract

The aim of this work is to present the method for sequential plutonium and americium activity determination in air filters using chromatographic radionuclide separation and alpha spectrometry measurement. The developed method may be employed for the purposes of workplace monitoring and as an indicator of the need of introducing the individual monitoring as well as a useful complementation of individual monitoring. Basic parameters describing the developed method such as values of chemical recoveries and minimum detectable activities for plutonium and americium isotopes have been determined. Applied counting efficiency was obtained using Monte Carlo calculation method.

## Introduction

Workers at nuclear facilities are exposed to internal contamination with radioisotopes. Thus, routine monitoring programme based on direct measurements (in vivo measurements) and the analysis of excreta samples (in vitro measurements) or samples taken from work environment (e.g., air) is carried out. Routine monitoring of people exposed to internal contamination at the nuclear facility at Otwock is performed by Radiation Protection Measurements Laboratory (LPD) of the National Centre for Nuclear Research.

From the point of view of radiological protection, analyses which can determine whether internal contamination with alpha emitters occurs are very important due to the fact that these isotopes are more radiotoxic and hazardous than others [1]. For this reason, routine monitoring (individual and of workplace) of the group of people working with alpha sources of ^238^Pu, ^239+240^Pu or ^241^Am is established. Commited effective dose values assessment is based on analyses of 24 h samples. Analyses of air activity, which are conducted within the framework of workplace monitoring, are considered to be complementation of the urinalyses [2]. In case the individual monitoring is not performed the workplace monitoring can provide data for doses estimation.

In considered situation the monitoring of air is realized by passing air aerosols through the Petryanov filter during the working hours. Measurement of filter activity is routinely performed in gas proportional counter. Laboratory recently developed the method for plutonium and americium activity determination in air aerosols collected on filters using radiochemical preparation and alpha spectrometry as a method of measurement. In the tested procedure for ^238^Pu, ^239+240^Pu or ^241^Am activity determination two types of resins were used: Dowex 1X8 and TRU. The effect of strong retention of Pu (IV) ions on Dowex 1X8 in 8 M HNO_3_ allows to perform plutonium separation. Isotopes of uranium and americium are not retained on the resin whereas thorium isotopes are stripped with 10 M HCl. Plutonium is eluted after its reduction to Pu (III). Separation of americium isotopes on TRU Resin is based on the fact that americium, as well as other actinides, shows high affinity for the resin for 1–3 M HNO_3_, however it is selectively eluted with 4 M HCl. The sources for alpha spectrometry measurements were prepared by electrodeposition of alpha emitting isotopes on steel discs. The sources prepared at LPD have a smaller diameter than the reference source of alpha emitters belonging to the laboratory. The influence of the source diameter on counting efficiency is significant. For that reason, Monte Carlo code MCNPX [3] was used to calculate the counting efficiency for samples prepared at the laboratory. In this paper this method is also shown. The aim of this work is to present adopted method on the basis of conducted analyses of air filters collected in 2014.

## Materials and methods

### Plutonium and americium tracer solutions

The laboratory used ^242^Pu and ^243^Am as yield indicators. ^242^Pu radioactivity standard solution was obtained from National Institute of Standards and Technology (NIST). ^243^Am was purchased from Amersham International. Solutions were diluted to specific activity of 0.1026 ± 0.0010 and 0.1013 ± 0.0020 Bq g^−1^, respectively.

### Chromatographic resins

The anionic resin Dowex 1X8 (100–200 mesh, chloride form) purchased from Sigma-Aldrich and glass columns of internal diameter of 10 mm were used for plutonium separation. For americium determination the extraction chromatography TRU Resin (100–150 µm) and 2 ml columns of 7 mm internal diameter obtained from Triskem International were applied.

### Equipment

Alpha activities of the samples were measured with Alpha Analyst Model 7200 spectrometer (Canberra, USA) with passivated silicon planar implanted detectors of 450 and 1200 mm^2^ effective area. Electrodeposition devices (electrodeposition cells, electrodes and power supplies) which were used during source preparation were described in a previous paper [4].

### Sampling

Static air sampler is used to carry out workplace monitoring. Air monitoring is realized by passing the aerosols from air through the filter during working hours. Applied filter is Petryanov Filter type FPP-15-1.5 (made of chlorinated polyvinylchloride) of 5 cm diameter. The filter is replaced once a week and then the activity of aerosols collected on the filter is measured. In 2014 employees were working with plutonium and americium isotopes for 21 weeks. Volume of air passing through the filters collected that year varied from 10 to 72 m^3^. All samples were measured with gas proportional counter. Nineteen air filters, available for laboratory, were analyzed by alpha spectrometry.

### Procedure

**Fig. 1:**
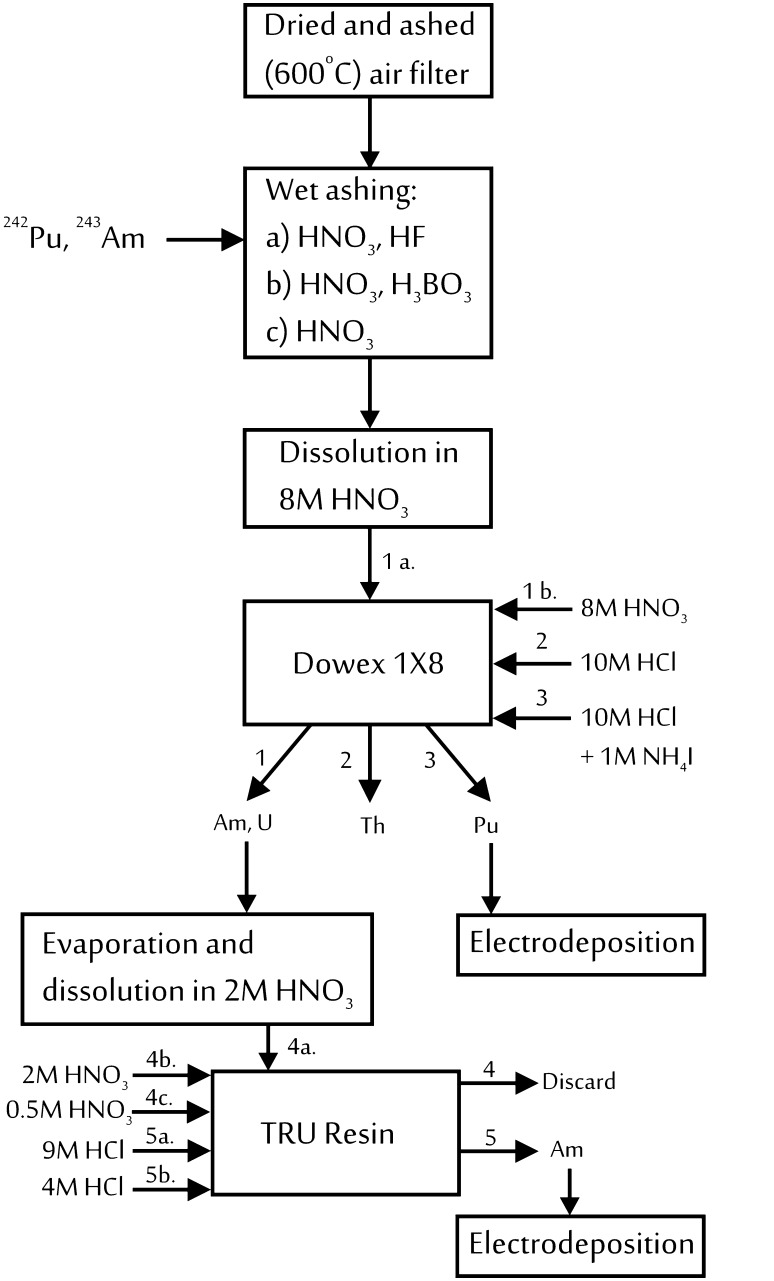
Sequential method for determination of plutonium and americium isotopes in air filters

Procedure for the sequential determination of plutonium and americium isotopes is schematically shown in Fig. 1. Detailed description of the presented method is given below.

#### Dry and wet ashing

At the first stage samples of air filters were dried, the mean mass of dried samples was 0.13 g. Dried sample of air filter was ashed in a muffle furnace (600 °C). After ashing sample was transferred using 10 cm^3^ of 65 % HNO_3_ to Teflon (PTFE) beaker. In order to destroy organic and silica matrix the combination of acids was used in the mineralization procedure [5]. The volume of 40 % HF added to each sample was 2.5 cm^3^. Before starting the wet ashing known activities of ^242^Pu and ^243^Am were added. The sample was mineralized for 3 h. After this stage sample became clear and then it was evaporated to dryness. In the next step 15 cm^3^ of 65 % HNO_3_ and 20 mg of H_3_BO_3_, which binds fluoride ions, were added and then evaporated. Nitric acid (11 cm^3^ of 65 % HNO_3_) was added to the sample, then the solution was boiled and transferred to a glass beaker. After evaporation, the residue was dissolved in 50 cm^3^ of 8 M HNO_3_.

#### Ion-exchange chromatography [6]

The bed height of Dowex 1X8 resin placed in a glass column was 9 cm. Resin was pre-conditioned with 50 cm^3^ of 8 M HNO_3_. Sample was loaded onto the resin after adjustment of plutonium valency (0.5 cm^3^ of 30 % H_2_O_2_, approximately 150 mg NaNO_2_). The resin was washed with 90 cm^3^ of 8 M HNO_3_. Sample and washing solution were collected for americium purification and subsequently evaporated. The second applied washing solution was 10 M HCl (100 cm^3^). This fraction was discarded. Finally, plutonium isotopes were eluted using a mixture of 10 M HCl (70 cm^3^) with 1 M NH_4_I (2 cm^3^).

#### Extraction chromatography [7]

TRU resin placed in a column was conditioned with 10 cm^3^ of 2 M HNO_3_. Residue obtained after evaporation of americium fraction was diluted in 20 cm^3^ of 2 M HNO_3_ and filtered. Then the sample was loaded onto the column and allowed to pass through the resin. Afterwards resin was washed with 10 cm^3^ of 2 M HNO_3_, 5 cm^3^ of 2 M HNO_3_ and 5 cm^3^ of 0.5 M HNO_3_, respectively. These fractions were discarded. To convert resin to chloride system 3 cm^3^ of 9 M HCl were added and next americium was eluted with 20 cm^3^ of 4 M HCl, these two fractions were collected in the same beaker.

Obtained solutions containing ^238^Pu, ^239+240^Pu and ^241^Am were evaporated and mineralized with 65 % HNO_3_ (2.5 cm^3^) and 35–38 % HCl (2.5 cm^3^). The method of sample preparation for the measurements in alpha spectrometer was electrodeposition [4]. The optimum conditions, determined for the electrodeposition of plutonium and americium are presented in Table 1. The counting time *t* of the prepared sources depends on plutonium and americium (^238^Pu, ^239+240^Pu or ^241^Am) activity. If the activities of the isotopes of interest were above MDA, the sample was measured until the uncertainty of peak area was below 10 %.

**Table 1:** Applied conditions (current and time) for plutonium and americium electrodeposition

	Time (h)	Current (A)
Plutonium	3	1
Americium	3	1.1

## Results and discussion

### Counting efficiencies

Calculation of sample activity does not require a knowledge of counting efficiency *ε*, while for the calculation of chemical recoveries *R* this value is needed. Many laboratories determine chemical recoveries because it is a suitable tool for checking the quality of performed analyses [8]. The counting efficiency depends not only on the sample—detector distance, but also the diameter of the source on which alpha emitting isotopes are deposited. The reference source diameter is 24.1 mm, while the prepared sources have a diameter of 20.0 mm. Counting efficiency for reference source is 20.1 ± 0.6 % (for detector of area of 450 mm^2^) and 32.5 ± 0.8 % (for detector of area of 1200 mm^2^) at the source to detector distance of 6.2 mm. Because of source diameter difference, the adoption of counting efficiency designated for the source of reference for further calculations is wrong, so one should find a value *ε* for sources made at the laboratory.

Efficiency calculations were usually performed using the Monte Carlo code MCNP [8]. In this case Monte Carlo simulation was also applied. It included a numerical model of the steel disc with a diameter corresponding to the diameter of the reference disc, and a model corresponding to the disc used at LPD (respectively 24.1 and 20.0 mm). The assumptions of the model:

- On the upper surface of the disc a surface source was placed emitting isotropically (at each point) alpha particles with energies corresponding to the energies of the reference source.
- The disc was placed in a test chamber filled with rarefied air at a pressure of 7 mmHg. At a distance of 6.2 mm a surface with a diameter corresponding to the diameter of the used detectors (39.1 and 23.9 mm) was defined.
- The calculations were performed for four possible combinations of the size of the disc and the detector.
- The model takes into account the resolution of energy detector and a window with a thickness of 500 Å, according to the manufacturer’s specifications [9].

The efficiency values determined based on the calculated spectra of alpha particles ($\varepsilon_{\text{MC}}$) using Monte Carlo code were compared to the values obtained in the measurement of the reference source ($\varepsilon_{\text{RS}}$) in alpha spectrometer by calculating their ratio (${{\varepsilon_{\text{MC}} } \mathord{\left/ {\vphantom {{\varepsilon_{\text{MC}} } {\varepsilon_{\text{RS}} }}} \right. \kern-0pt} {\varepsilon_{\text{RS}} }}$). The ratio values is very close to 1, indicating very good consistency of the efficiency values. Comparison of the results is presented in Table 2.

**Table 2:** Comparison of counting efficiency obtained by calculation method and reference source measurements

Detector	Source	${{\varepsilon_{\text{MC}} } \mathord{\left/ {\vphantom {{\varepsilon_{\text{MC}} } {\varepsilon_{\text{RS}} }}} \right. \kern-0pt} {\varepsilon_{\text{RS}} }}$
Area 450 mm^2^ Diameter 23.9 mm	Reference	1.02
Area 1200 mm^2^ Diameter 39.1 mm	Reference	1.01

In further calculations, the following counting efficiencies values for sources of 20.0 mm diameter, resulting from the application of Monte Carlo code MCNPX, were taken:

- for 450 mm^2^ detector *ε* = 23.1 ± 0.7 %,
- for 1200 mm^2^ detector *ε* = 33.6 ± 0.8 %.

### Radiochemical recoveries

**Fig. 2:**
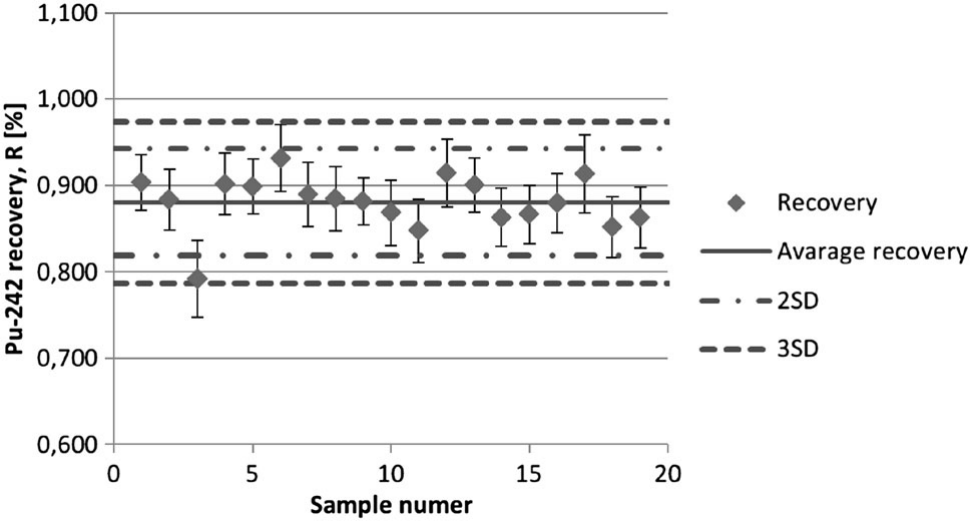
^242^Pu recoveries from analysed air filters

**Fig. 3:**
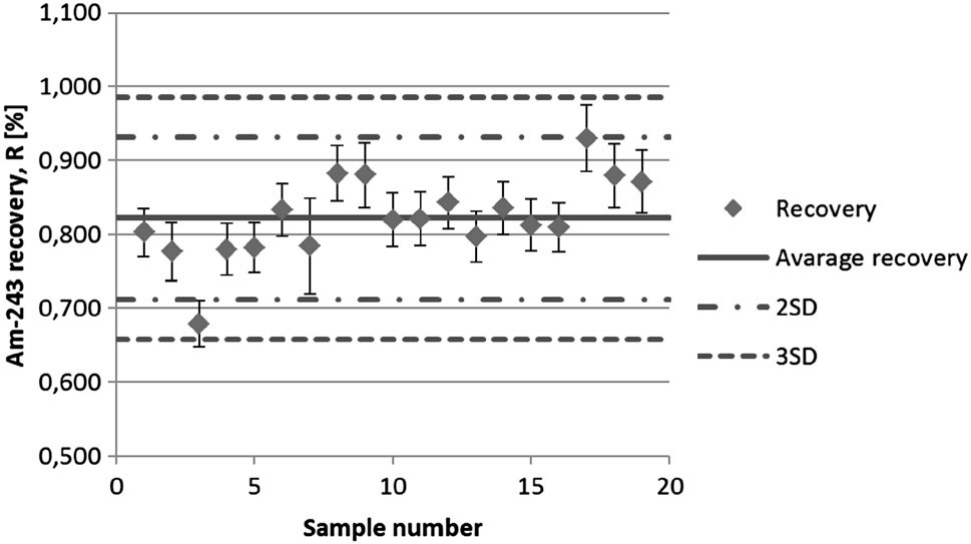
^243^Am recoveries from analysed air filters

The radiochemical recoveries of the procedure were determined by the addition of ^242^Pu and ^243^Am tracers to the samples. The activity of tracers spiked into the samples was in the range 10–12 mBq. Based on alpha spectra of measured samples the values of chemical recoveries, which are presented in the Figs. 2 and 3, were calculated. The average radiochemical tracer recoveries obtained for ^242^Pu and ^243^Am are 88 ± 3.1 and 82 ± 5.5 %, respectively. In one case when peak area of ^242^Pu was significantly smaller than peak area of ^238^Pu (sample number 9) it was impossible to calculate tracer recovery for ^242^Pu. In this situation mean value of radiochemical recovery, obtained for eighteen samples, was taken into calculation of plutonium activity.

### Minimum detectable activity

Values of minimum detectable activity (MDA) were calculated using the following equation [10]:

1 $$MDA = \frac{4.66 \cdot \sqrt B + 3}{t \cdot \varepsilon \cdot R}.$$

In this equation parameter *B* means counts of background. Table 3 shows the range of obtained values of MDA and parameters applied in calculations. Different values of MDA were caused mainly by differences in counting time depending on the sample activity.

**Table 3:** MDA values of the procedure for Pu and Am activity determination

Isotope	Counting time, *t* (s)	Background counts, *B* (–)	Recovery, *R* (–)	Counting efficiency, *ε* (–)	MDA (Bq sample^−1^)
^239+240^ Pu	18,277–962,186	0–9	0.79–0.93	0.231 and 0.336	0.0001–0.001
^238^Pu	18,277–962,186	0–9	0.79–0.93	0.231 and 0.336	0.0001–0.001
^241^Am	87,594–622,196	0–6	0.68–0.93	0.231 and 0.336	0.0001–0.0004

### Alpha spectra

**Fig. 4:**
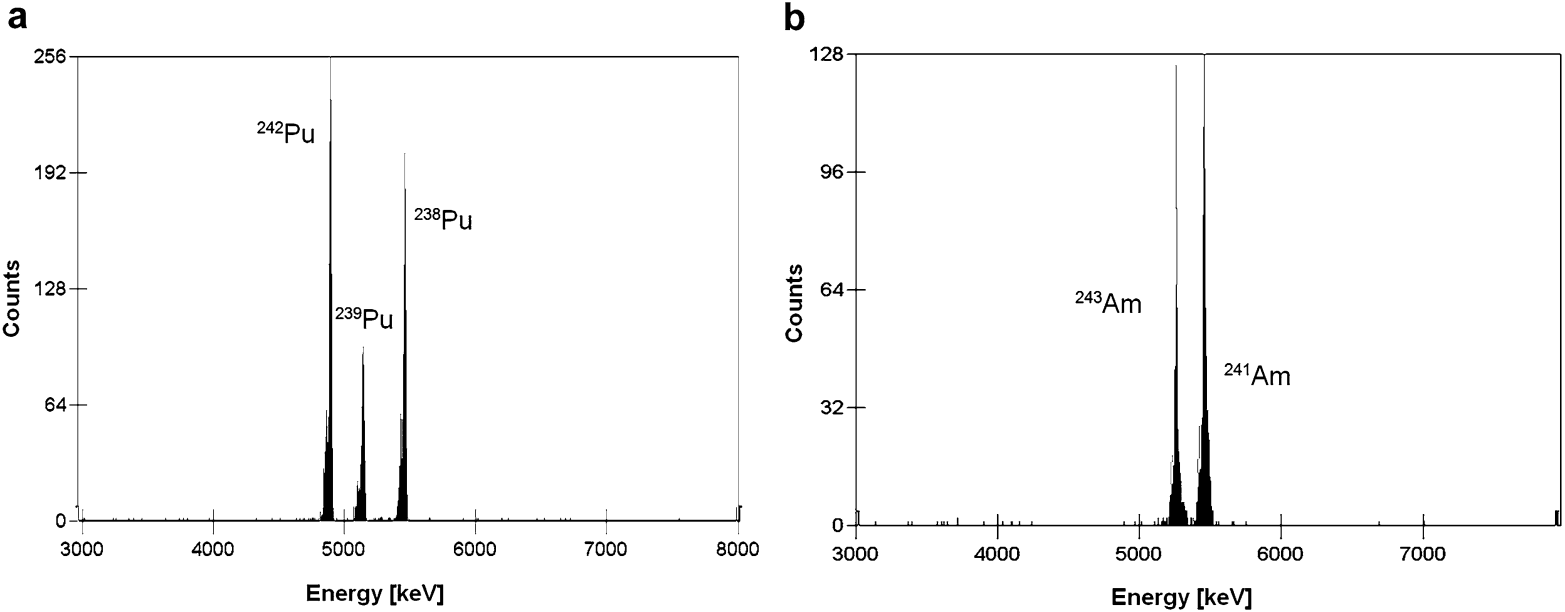
Alpha spectra of plutonium (**a**) and americium (**b**) isotopes from air filters

A very important aspect in the radiochemical preparation, that precedes alpha spectrometry measurements, is to obtain purified and thin sources. Otherwise, some isotopes can interfere with determined element and degradation of alpha spectrum parameters (such as FWHM—full width at half maximum) is observed [11]. Figure 4 relates to alpha spectra of plutonium (a) and americium (b) isotopes detected in one of analysed samples. The obtained peaks are characterized by good resolution and interferences are not observed.

### Samples measurements

In spite of the fact, that people working with alpha emitting isotopes used glove boxes, plutonium and americium isotopes occurred in air. The results obtained based on performed analyses show low activity concentration of ^238^Pu, ^239+240^Pu and ^241^Am in air aerosols (Table 4).

**Table 4:** Results of performed analyses of air filters collected in 2014

Isotope	Sample number	Maximum activity (mBq sample^−1^)	Maximum activity concentration (mBq m^−3^)
^239+240^Pu	7	14 ± 1.1	0.25 ± 0.019
^238^Pu	9	230 ± 23	10 ± 1.0
^241^Am	7	120 ± 19	2.0 ± 0.33

The main aim of analyses carried out was to estimate effective doses *E* for employees who worked with ^238^Pu, ^239+240^Pu or ^241^Am. If the effective dose is calculated according only to analyses of the activity of aerosols in the air, the following equation will be used:

2 $$E = \sum_iC_{\text{m,i}} \cdot \Delta T \cdot e\left( g \right)_{\text{i}}\,\cdot\,S.$$

Where: *C*_m,i_ is the measured concentration of radionuclide *i* (Bq m^−3^); $\Delta T$ time of work (h); *e*(*g*)_i_ the committed effective dose per unit intake for inhalation (Sv Bq^−1^); *S* lungs ventilation per hour during work activities for worker (m^3^ h^−1^). Reference values of breathing parameters given by ICRP [12] are usually taken. Values of effective doses, calculated using the values of activity concentrations shown in Table 4, derived from intake of ^239+240^Pu, ^238^Pu, ^241^Am during the working week by one employee are 0.24, 7.3 and 1.7 µSv, respectively.

According to recommendations [2, 13], effective doses should be estimated using the results of bioassay measurements, if they were performed. Data from air monitoring should complement individual monitoring [14]. Workplace monitoring enables the verification of the route of intake. It is an important aspect because workers often are contaminated via ingestion, while they touch the mouth with their hands at workplace [13].

Special attention should be paid to the situation when as a result of urine analyses contamination is not detected, while airborne monitoring, can provide information about air contamination. In such case the intake is too low to be detected by analyses of urine samples thus workplace monitoring can be useful for the assessment of low exposure.

## Conclusions

This paper refers to the tested method for ^238^Pu, ^239+240^Pu and ^241^Am activity determination in air filters and analyses conducted in 2014. Developed method using radiochemical preparation and alpha spectrometry is characterised by high chemical recovery and low MDA. The values of counting efficiencies resulting from Monte Carlo code MCNPX simulation were applied in the calculation of the chemical recoveries. As a result of radiochemical procedure purified alpha sources were obtained. Performed analyses confirm the presence of low activities of ^238^Pu, ^239+240^Pu and ^241^Am in air from the laboratory where the work with alpha sources takes place.

Analytical and measurement method for plutonium and americium determination is considered appropriate for radiological monitoring of the air in workplaces. On the basis of performed air filters analyses and urinalyses of workers exposed to the risk of internal contamination by ^238^Pu, ^239+240^Pu and ^241^Am it is possible to evaluate the effective dose more precisely. Results of urinalyses will be the subject of further studies.
